# Detection and Comparison of Bioactive Compounds in Different Extracts of Two Hazelnut Skin Varieties, Tonda Gentile Romana and Tonda Di Giffoni, Using a Metabolomics Approach

**DOI:** 10.3390/metabo11050296

**Published:** 2021-05-05

**Authors:** Veronica Lelli, Romina Molinari, Nicolò Merendino, Anna Maria Timperio

**Affiliations:** Department of Ecological and Biological Sciences, University of Tuscia, 01110 Viterbo, Italy; v.lelli@unitus.it (V.L.); molinari@unitus.it (R.M.); merendin@unitus.it (N.M.)

**Keywords:** hazelnut skin, untargeted metabolomic, phenolic compounds, nutraceuticals

## Abstract

Agro-wastes are one of the major sources for nutritional and therapeutic benefits along with other beneficial properties. Dark brown pellicular pericarp (skin or testa), covering the hazelnut seed, is removed before consumption after the roasting of a kernel. Defatted skins of both hazelnut varieties, *Tonda Gentile Romana and Tonda di Giffoni*, were profiled by a metabolomics-based approach and this was used to discriminate between these two different hazelnut cultivars. In particular, an untargeted metabolomic extract from hazelnut by-products was investigated by UHPLC-Mass spectrometry followed by multivariate statistics analysis, and significant qualitative and quantitative metabolic differences were observed between them. Samples were also assessed for their total phenolic and antioxidant capacity using two different assays. Although no significant differences were found in total phenolic contents and antioxidant capacity, the Flavone, Flavonol, Flavonoid, and Phenylpropanoid Biosynthesis pathway was significantly higher in the *Romana* rather than in the *Giffoni* variety, whereas Myricetin and Syringetin compounds were more representative in *Giffoni* cultivars. These results indicated that hazelnut skin, especially from the Romana variety, could potentially be used as an ingredient in healthy food. Healthy food is a new food category with an expanding demand from future generations.

## 1. Introduction

A sustainable future for the human race must include the effective reuse and recycling of waste streams. Wastes are generally considered worthless at the time of production, yet they can still contain resources that may become valuable [1]. In the whole food production chain, from industry to the fork, food manufacturing steps have a large environmental impact, as ∼30–50% of produced foods become waste [2]. The development of green technologies in the food manufacturing sector is particularly relevant with the objective to convert raw agro materials into food products with the desired quality and functional properties while increasing manufacturing efficiency [3]. The wastes generated from the processing of various plant-based foods are rich in sugars, pectin, proteins, lipids, polysaccharides, fibers, flavor compounds, and phytochemicals. Such value-added products have immense value as food additives, nutraceuticals, therapeutics, cosmetics, etc. [4,5]. Natural ingredients recovered from agro-industrial by-products have specific dietary and functional properties and can be utilized effectively to develop this new food category. In this context, we considered them in our manuscript hazelnut. This nut belongs to the family Betulaceae [6], and was determined to be a “heart-healthy” food [7] by Food and Drug Administration (FDA) and recommended in a balanced diet due their health-promoting properties. The seed is covered by a dark brown pellicular pericarp (skin or testa) [8] and is marketed as natural and processed kernels. Whole processed hazelnut kernels, which are called roasted hazelnuts, constitute the largest quantity of hazelnut consumption [9,10]. Hazelnut skin, which represents about 2.5% of the total hazelnut kernel weight, is removed during roasting, and has very low economic value, has traditionally been used for livestock feed and as a raw material for energy production. Hazelnut skins have a significant portion of polyphenols that can have covalent or strong non-covalent associations with plant proteins and polysaccharides [11]. Moreover, hazelnut skin is a rich source of dietary fiber as well as of natural antioxidants owing to the presence of phenolic compounds [6]. Recent research by Ozyurt et al. [10], determined total phenolic content to identify the total antioxidant activity to characterize the phenolic profile of hazelnut skin. Additionally, Contini et al. [12] verified the presence of tannin compounds obtained from phenolic extracts of hazelnut shell and skin wastes. Therefore, the usage of hazelnut skin as a source of antioxidants and dietary fiber may offer beneficial supplements to the food, medicine, and cosmetic industries [7]. The phenolic compounds play a role in human health promotion, disease risk reduction [13,14], and providing protection against the harmful effects of free radicals, which impose the risk of cancer, stroke, inflammation, and other neurodegenerative diseases [15,16]. Indeed, hazelnut has a clear territorial characterization, and are redistributed in a few countries [17]. Hazelnut is a nut species cultivated in temperate climates such as those of the Black Sea coast of Turkey, hilly areas of Italy, northern regions of Spain, and Willamette Valley in Oregon, USA [18]. A mild climate, high rainfall, and cool summers are favorable characteristics and Italian varieties are currently qualified for protected geographical indication (PGI) (i.e., ‘Tonda Gentile delle Langhe’ e ‘Tonda Gentile Romana’) or protected designation of origin (PDO) (‘Tonda di Giffoni’) certifications. Italy is the second largest producer of hazelnuts (13%) after Turkey [19]. Italy’s Latinum regions contain more than 25% of the national production areas, concentrated in the Viterbo province as a typical hazelnut-growing area. Current research highlights the utility of metabolomics as high-throughput methods to profile variations in nutrients and bioactive compounds in foods. In the last few years, metabolomics has emerged as a new powerful approach, focusing on the comprehensive analysis of small-molecule metabolites in biological systems [20]. Moreover, multivariate analysis on metabolomics-based data allows gaining information directly correlated to food quality, safety, processing, storage, and authenticity assessments [21]. Hence, this study aimed to evaluate the differences between two varieties of hazelnut skin through a procedure able to detect a wide variety of metabolites from *Tonda Gentile Romana* and *Tonda di Giffoni* varieties coming from our geographic area. To ensure broad metabolite detection coverage on skin samples, we employed hydrophilic interaction liquid chromatography (HILIC), electrospray ionization-mass spectrometry (ESI-MS), a technology particularly suitable to separate samples and complex mixtures. Applying this experimental approach, one could identify the metabolites that characterize the two varieties.

## 2. Results and Discussion

The main purpose of this work was to analyze the global metabolite profile of skin coming from the industrial pericarp removal during the processing of whole or chopped hazelnut kernels. To achieve this aim, we analyzed the phenolic profiles and total antioxidant activities in two varieties of hazelnut by-products: *Tonda Gentile Romana and Tonda di Giffoni*. The content of total phenols ranged from 155.3 ± 2.1 mg GAE /g DW in Giffoni hazelnut skin to 153.3 ± 3.9 mg GAE /g DW in Romana hazelnut skin; the content of total flavonoids ranged from 27.2 ± 0.7 mg RE /g DW in Giffoni skin to 27.8 ± 2.3 mg RE /g DW in Romana skin (Table 1). Del Rio and coworkers (2011) [22] reported that the phenol content in aqueous extracts was 8.7 ± 0.3 g/100 g in Giffoni skin and 9.0 ± 0.0 in Romana skin; Contini and collaborators (2008) [12], showed that phenol content in methanol extract carried out on different varieties (Italian Tonda Gentile Romana, Tonda di Giffoni, Tonda Gentile delle Langhe, Turkish Tombul) was 426.7 ± 4.6 mg GAE/g. Since the antioxidant compounds in foods are chemically different and structurally complex, no single assay is suitable to accurately determine the antioxidant activity for all compounds. For this reason, antioxidant activity was determined in our study using two different antioxidant assays: FRAP and TEAC ABTS. The FRAP assay is based on an electron transfer mechanism without the involvement of free radicals, while TEAC ABTS assay is based on the conversion of oxidized ABTS^•^^+^ radicals to ABTS via a hydrogen atom transfer (HAT) or single electron transfer (SET) mechanism. The antioxidant capacity ranged from 13.2 ± 0.1 mM TE in Giffoni hazelnut skin to 13.1 ± 0.2 mM TE in Romana hazelnut skin with the TEAC method and to 23.3 ± 5.8 mM TE in Giffoni skin to 23.1 ± 6.2 mM Te in Romana skin with the FRAP method (Table 1). Contini and collaborators (2008) [12], showed that the antioxidant capacity in methanol was 68 ± 1.2 mg GAE/ mg DPPH); Del Rio and coworkers (2011) [22] reported that antioxidant capacity was 1321.1 ± 54.4 mmol Fe^2+^ Equivalent/kg in Giffoni and 1229.9 ± 47.0 Fe^2+^ Equivalent /kg in Romana. The difference in phenolics content and antioxidant capacity could depend on the extraction methods/solvents used and on environmental factors. The proximate composition of hazelnut skin is given in Table 2. Moisture, protein, and ash contents of the skins are shown on a dry weight (dw) basis and they are similar to reported data [23]. The lipid content is in the range of 18.4–26.4% from Giffoni skin (18.4%) to Romana skin (26.4%). Carbohydrates are the major components of skin; these results are in agreement with other studies [23]. Our results showed that the total phenols and flavonoids between varieties do not undergo variations, any difference likely lies in the phenol types. To verify the metabolomic differences between the varieties and eventually to the characterized phenol compounds, a more sensitive method consisting of UHPLC system coupled online with a Q Exactive mass spectrometer analysis was performed following a statistical multivariate analysis. Because significant amounts of data are obtained through metabolomic analysis, they must be interpreted. Chemometric methods help manage this enormity of information. For this purpose and to highlight the most significant differences among the metabolites between the two groups, we performed statistical analyses through Metaboanalyst v 4.0 (Wishart research Group at the University of Alberta, Edmonton, AB, Canada).

**Figure 1:**
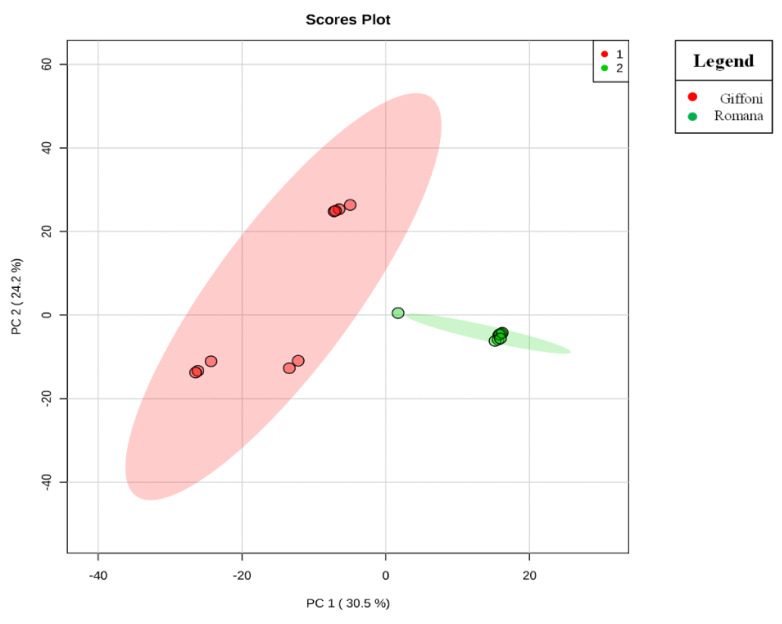
Principal component analysis (PCA) scores plot. The PCA scores plot shows a clear segregation of Tonda di Giffoni and Romana samples, indicating that Tonda di Giffoni and Tonda Gentile Romana have different metabolic profiles.

**Figure 2:**
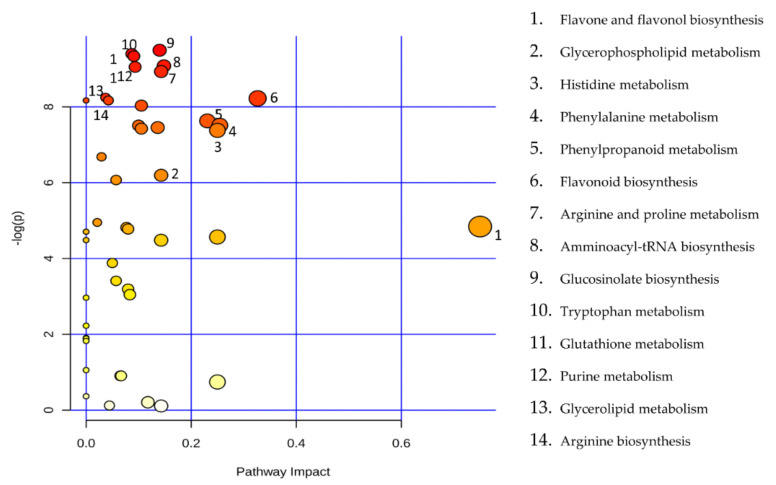
Metabolic Pathway Analysis (MetPA). All the matched pathways are displayed as circles. The colour and size of each circle are based on the *p*-value and pathway impact value, respectively. The graph was obtained by plotting the −log of *p* values from the pathway enrichment analysis on the *y*-axis and the pathway impact values derived from the pathway topology analysis on the x-axis.

**Figure 3:**
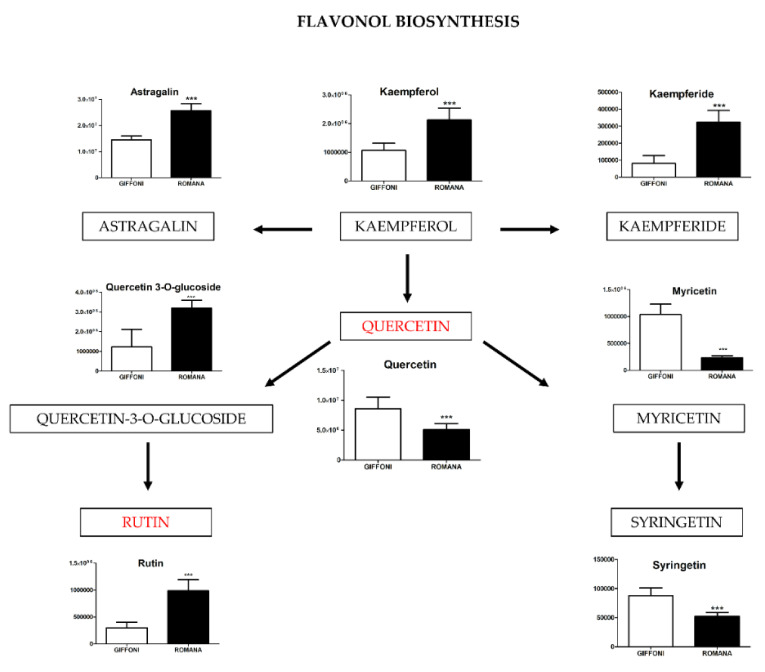
The level of metabolites involved in the Flavonol biosynthesis were analyzed in Giffoni and Romana hazelnut skin. Values are presented as the mean ± SD. *** (*p* ≤ 0.001).

**Figure 4:**
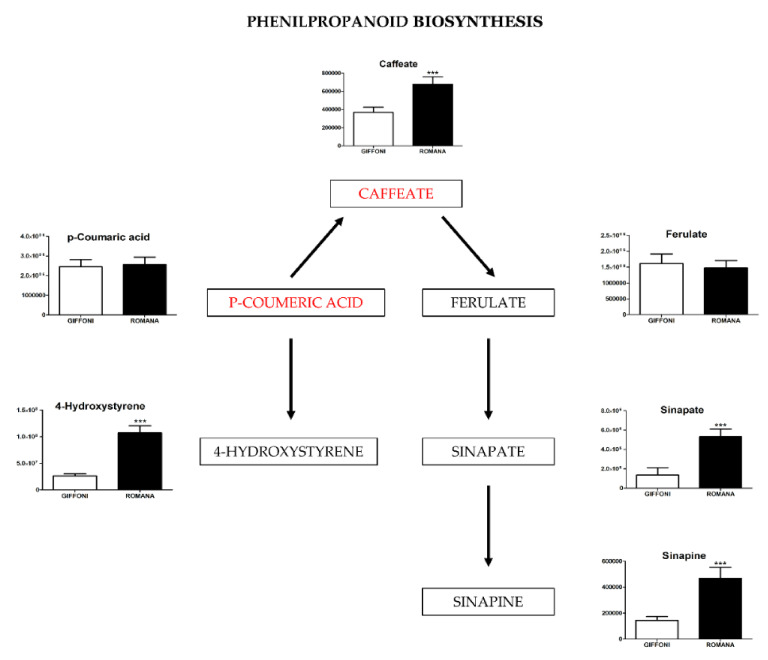
The figure represents metabolites involved in the Phenilpropanoid biosynthesis found in different concentrations in the two varieties of hazelnut skin. Data are presented as the mean ± SD. *** (*p* ≤ 0.001).

The principal component analysis (PCA) showed a clear separation of Romana from Giffoni skin (Figure 1), with 57.6% of the variance captured by the first two PCs. To confirm PCA results with a more powerful pattern recognition method, we performed a supervised PLS-DA (Partial Least Squares Discriminant Analysis). Satisfactory modeling and prediction results were already gained with three PCs (Accuracy 0.94444, R^2^ > 0.99944, Q^2^ > 0.93344) (Supplementary Materials Figure S1). Metabolomic analysis was conducted through two strategies that are complementary to each other. The first gave overall metabolic changes without going to analyze the concentrations of the metabolites present to obtain a global view. Table 3 obtained by using MAVEN lists the major metabolites differentially expressed in the two *Tonda Gentile Romana* and *Tonda di Giffoni* varieties. MAVEN is an open-source cross-platform metabolomics data analyzer. The purpose of this software package is to reduce the complexity of metabolomics analysis by incrementing a high instituted interface for examining and validating metabolomics data. The program features multi-file chromatographic aligners, peak-feature detector, an isotope and adduct calculator, formula predictor, pathway visualizer, and isotopic flux animator. From this table, several compounds were identified such as flavonoids (eriodictyol, quercetin, kaempferol, rutin), phenolic acids (neochlorogenic acid, caffeic acid, coumaric acid) flavan-3-ols (catechin, myricetin) and other compounds, these results are comparable to those obtained by Del Rio [22]. Moreover, MAVEN supports data from ESI-MS. To better identify which metabolic pathways were most affected in these two species, we used the bioinformatics Metabolomics Pathway Analysis (MetPA) by MetaboAnalyst 4.0, which combines the results from the pathway enrichment analysis with the pathway topology analysis (Figure 2). The “metabolome overview” obtained through metabolic pathway analysis (MetPA) showed that the Flavonoid and Phenylpropanoid biosynthesis were significantly perturbed (FDR < 0.05; pathway impact value > 0.2) between *Tonda Gentile Romana* and *Tonda di Giffoni variety* and we focused our attention on these two pathways. To this aim, we applied a second investigation strategy called “metabolic profiling” that allowed us to conduct qualitative and quantitative analysis of the individual metabolites. Figure 3 shows the quantitative and qualitative variations among metabolites belonging to the biosynthesis of flavonoids between the two varieties. Quercetin, which is the main representative metabolite of the Flavonol Biosynthesis, was significantly increased in Giffoni skin. Quercetin, the aglycone structure of rutin, is one of the most prominent dietary antioxidants; quercetin has many molecular and physiological effects in various organisms, including humans; it may have key roles in combating asthma, diabetes, obesity, cardiovascular, neurological, and inflammatory diseases [24] and cancer [25]. Due to the biological and pharmacological importance of quercetin as a bioactive flavonoid, we commonly consider the Giffoni skin to be more useful for sources of nutritional and therapeutic purposes. Quercetin is present in plants in many different glycosidic forms. It is usually found in conjugated forms with sugars such as glucose, galactose, and rhamnose [26]. Rutin, chemically, is a glycoside composed of the flavonol aglycone quercetin along with the disaccharide rutinose. The “metabolic profiling” in Figure 3 showed that rutin was significantly increased in Romana skin. Rutin has demonstrated a number of pharmacological activities, including antioxidant, cytoprotective, vasoprotective, anticarcinogenic, neuroprotective, and cardioprotective activities [27,28,29,30,31,32,33,34]. Rutin which is one of the best natural antioxidants in the known natural class, is absorbed more slowly than quercetin because it must first be hydrolyzed by the gut microbiota, whereas quercetin is absorbed from the small intestine. The prevalent forms are quercetin conjugated with one or two glucose molecules, such as isoquercetin and quercetin conjugated with rutinose such as quercetin, rutinoside rutin, and quercetin 3-o-glucoside that we found to be significantly upregulated in Romana species (Figure 3). Moreover, quercetin 3-o-glucoside exhibits more anti-inflammatory action than other forms of quercetin [35]. Thus, the higher level of these two metabolites shown in Romana cultivars led to believe that this species could have more healthy properties than Giffoni. Besides the biosynthesis of flavonoids, the phenylpropanoid biosynthesis is altered between the two varieties. In addition, in this case in the Romana variety, the intermediates of the metabolism of phenylpropanoids were found to be increased, as shown in Figure 4. Many biological activities of Phenylpropanoid biosynthesis (Figure 4) are attributed to ester derivatives of caffeic acid (strongly active in Romana) such as anti-inflammatory, anti-cancer by induction of apoptosis, antimicrobial, and immunomodulatory activity. Several studies have shown that caffeic acid and ferulic acid can inhibit the oxidation of low-density lipoprotein (LDL) in vitro [36,37,38]. Furthermore, caffeic acid is absorbed in rats and has an absolute bioavailability in humans of around 95% [39,40]. Finally, appropriate model validation is a crucial step to assess reliability for quantitative or confirmatory purposes. Based on these results, we moved from targeted to untargeted mass spectrometric methods and we quantified the principal phenolic compounds of phenylpropanoid pathways using authentic standards (Table 4) to demonstrate if they were effectively present in quantities that enable consideration of hazelnut skin as a beneficial food for health. Notably, the quantity of the flavonoids detected is remarkably high, enabling hazelnut skin to be considered useful as an antioxidant. The concentration of quercetin is higher in the Giffoni, while the concentration of rutin is higher in the Romana. In particular, the antioxidant capacity of various nuts and their byproducts has been widely investigated, and several works have acknowledged that nut byproducts are especially rich sources of natural phenolic compounds with potential bioactivities [41,42]. Here, we considered two local varieties in an attempt to add economic value to waste from the hazelnut industry. However, we also consider it necessary to examine the bioavailability of these compounds after the digestion process, with the aim of clarifying the effective bioavailability of the hazelnut skin constituents (paper in preparation).

## 3. Materials and Methods

### 3.1. Raw Materials and Extract Preparation

Hazelnut skins (Tonda Gentile Romana and Tonda di Giffoni variety) were obtained from a local producer (Bionocciola srl, Carbognano, Viterbo, Italy) after roasting hazelnuts at 150 °C for 24 min. To measure the antioxidant activity, total polyphenol, and total flavonoid contents, 0.5 g of two varieties of Tuscia hazelnut skins were extracted with a solvent consisting of methanol:water (80:20, *v*/*v*) in a ratio 1:20. A total of 20 mg of two varieties of Tuscia hazelnuts skins were used for metabolomics analysis. The 1:25 mixture was shaken at room temperature for 2 h, and then centrifuged at 1000× *g* for 10 min, the supernatant was used for all analyses. All chemicals used were of analytical grade and were purchased from Sigma Aldrich (Milano, Italy). Stock standard solutions of phenolic compounds were prepared by rigorous dissolution of the commercial reagent in methanol. High-purity water from a Direct-Q 3 UV water purification system (Millipore, Burlington, MA, USA) was used for all analyses. 

### 3.2. Determination of Total Phenols

Total phenols were determined using a modification of the Folin and Ciocalteu standard method [43]. Briefly, the assay was conducted by mixing 4 mL of deionized water, 0.25 mL of extract, 0.25 mL of Folin Ciocalteu reagent, and 0.5 mL of Na_2_CO_3_. After employing the Singleton pattern for 30 min at room temperature, the absorbance of the mixture was measured on the Uvikon spectrophotometer (942, Kontron Instruments, Zurich, Switzerland) at 725 nm. A standard curve with gallic acid was prepared. The final results were expressed as mg of gallic acid equivalents (GAE)/ g of dry weight (DW). All analyses were conducted in nine replicates. 

### 3.3. Determination of Total Flavonoids

The total flavonoid contents in the samples were determined using the aluminum chloride colorimetric method described by Qin et al. [44]. Briefly, the appropriate dilutions of the extract (0.5 mL) were mixed with 1.5 mL of 95% ethanol, 0.1 mL of 10% aluminum chloride hexahydrate (AlCl_3_×6H_2_O), 0.1 mL of 1 M potassium acetate (CH_3_COOK), and 2.8 mL of deionized water. After incubation at room temperature for 30 min, the absorbance of the reaction mixture was measured at 415 nm with Uvikon spectrophotometer (942, Kontron Instruments, Zurich, Switzerland). The total flavonoid contents were expressed as mg rutin equivalents (RE)/g DW. Samples were analyzed in nine replicates. 

### 3.4. Measurements of Total Antioxidant Capacity Using FRAP and TEAC Assay

A ferric reducing antioxidant power (FRAP) assay was performed according to the method described by Benzie and Strain [45], which was adapted for 96-well plates and an automatic reader. The method is based on the reduction of the Fe^3 +^ -2,4,6-tripyridyl-s-triazine (TPTZ) complex to its ferrous form at low pH. Briefly, 160 μL of FRAP assay solution (consisting of 20 mM ferric chloride solution, 10 mM TPTZ solution, and 0.3 M acetate buffer at pH 3.6) was prepared daily, mixed with 10 μL of the sample, standard, or blank, and dispensed into each well of a 96-well plate. The absorbance was measured at 595 nm at 37 °C after 30 min of incubation. All analyses were conducted in nine replicates. The final results are expressed as mM Trolox equivalents per g of the DW samples (mM TE/g DW). The TEAC assay (Trolox Equivalent Antioxidant Capacity) was performed using the OxiSelect TEAC Assay Kit (Cell Biolabs, San Diego, CA, USA) following the company protocol. 

### 3.5. Proximate Composition

Moisture, ash, as well as protein, lipid, and carbohydrate contents in skin samples were determined according to the standard procedures of the Association of Official Analytical Chemists International [46]. The moisture and ash contents were determined using standard methods [45,46]. Total nitrogen and total protein contents (conversion factor: 6.25, AOAC 2007, 2001.11) were obtained using Kjedahl digestion (BUCHI Auto Kjeldahl Unit K370 and BUCHI Speed Digester K439). Lipid fraction was extracted using a semiautomatic Soxhlet Buchi Extraction System B-811 (Buchi Labortechnic AG, Flawil, Switzerland) with petrol ether as the extraction solvent (AOAC 2007, 920.39). Carbohydrate content was calculated by subtracting the contents of other compositions from 100%. 

### 3.6. Analysis of Phenolic Compound Composition by LC-MS

Untargeted metabolomics was applied to investigate the biochemical alterations of hazelnut skin. A total of 20 microliters of the extracted samples was injected into an Ultra High-Performance Liquid Chromatography (UHPLC) system (Ultimate 3000, Thermo, Fisher Scientific, Waltham, MA, USA), which was operated in positive ion mode. A Reprosil C18 column (2.0 mm × 150 mm, 2.5 μm-Dr, Maisch, Germany) was used for metabolite separation. Chromatographic separations were made at a column temperature of 30 °C and a flow rate of 0.2 mL/min. For positive ion mode (+) MS analyses, a 0–100% linear gradient of solvent A (ddH_2_O, 0.1% formic acid) to B (acetonitrile, 0.1% formic acid) was employed over 20 min, returning to 100% A in 2 min and holding solvent A for a 6-min post-time hold. Acetonitrile, formic acid, and HPLC-grade water and standards (≥98% chemical purity) were purchased from Sigma Aldrich. The UHPLC system was coupled online to a Q Exactive mass spectrometer (Thermo) scanning in full MS mode (2 μscans) at a resolution of 70,000 in the 67 to 1000  *m*/*z* range, a target of 1106 ions and a maximum ion injection time (IT) of 35 ms with 3.8 kV spray voltage, 40 sheath gas, and 25 auxiliary gas. The system was operated in positive ion mode. Calibration was performed before each analysis with positive or negative ion mode calibration mixes to ensure error of the intact mass within the sub ppm range. Metabolite assignments were performed using computer software (Maven 18 Princeton, NJ, USA), upon conversion of raw files into a mzXML format using MassMatrix (Cleveland, OH, USA). MAVEN is an open-source software that could be freely downloaded from the official project website. To further explore the metabolic differences between the two cultivars, multivariate statistical analyses were employed using the MetaboAnalyst 4.0 software. Before the analysis, the raw data were normalized by sum and auto-scaling to increase the importance of low-abundance ions without a significant amplification of noise. The web-based tool MetPA (Metabolomic Pathway Analysis), which is incorporated into the MetaboAnalyst platform, was used to perform pathway analyses. Data for metabolites detected in all samples were submitted to MetPA with annotations based on common chemical names. Global test was the selected pathway enrichment analysis method, whereas the node importance measure for topological analysis was the relative betweenness centrality. Metabolites subject to major changes were graphed with Graphpad Prism 5.03 (Graphpad SoftwareInc, San Diego, CA, USA, 92108). Statistical analyses were performed with the same software. Data are means of nine replicates ± SD. Differences were considered statistically significant at * *p* < 0.05 and further stratified to ** *p* < 0.01 and *** *p* < 0.001, respectively. For the analysis of antioxidant capacity, total phenol and flavonoids data are presented as mean ± standard deviation of nine independent replicates. Statistical comparisons were made by one-way analysis of variance followed by Fisher’s comparison test using the XLSTAT 2019 software (Addinsoft SARL, New York, NY, USA). Values were considered significant when *p* < 0.05 [r1].

### 3.7. Quantitative Analysis

The flavonols were identified on the basis of their MS spectra and molecular-ion identification. Quantification of these compounds was calculated relative to the corresponding external standards from the calibration curves that covered the range from 0.002 mg/mL to 0.2 mg/mL. Data are presented as the mean ± SD.* (*p* ≤ 0,05). For an initial evaluation of the calibration function, as part of the method validation, standards with different concentrations (including a blank) were prepared. The calibration range was established so that most sample concentrations fall within the range. Each study sample is divided into aliquots of equal volumes and known and variable amounts of the standard were added to the aliquots to construct the calibration curve. The calibration solutions were prepared from a pure substance with a known purity value. Commercial Standards: Quercetin ≥95% (HPLC), Rutin ≥94% (HPLC), p-Coumaric acid ≥98% (HPLC), Vitexin ≥95.0% (HPLC), and Caffeic Acid ≥98% (HPLC), were purchased from Sigma-Aldrich Co. (St. Louis, MO, USA).

## 4. Conclusions

In conclusion, we investigated the composition of byproducts of two hazelnut local varieties: *Tonda Gentile Romana* and *Tonda di Giffoni*, in an attempt to add economic value to waste from the hazelnut industry that could transform this waste into a precious renewable and natural resource. Metabolomics data revealed that Giffoni and Romana have distinct metabolic profiles. One of the significant differences between the two profiles was the polyphenols compounds, in particular Flavones, Flavonol, Flavonoid, and Phenylpropanoid biosynthesis, were found to be higher in Romana varieties, which supports the idea of the potential for new targets for health. Flavonoid and Phenylpropanoid biosynthesis pathways are significantly higher in Romana rather than in Giffoni varieties, whereas Myricetin and Syringetin compounds are more representative in Giffoni cultivars. Our results suggest that hazelnut skin can be used in the preparation of low calorie, high fiber, and antioxidant-rich foods, as well as food supplements, and active pharmaceuticals. Finally, the manuscript provides for the first time some new information regarding flavonoids in these hazelnut skins using LC-MS.

## Figures and Tables

**Table 1:** TE, GAE and RE of hazelnuts skin. Total antioxidant capacity of hazelnut skins (TE Trolox equivalent), total polyphenols contents (GAE Gallic Acid Equivalent), total flavonoids contents (RE Rutin Equivalent).

Varieties	Antioxidant Capacity		Polyphenols	Flavonoids
	TEAC mM TE	FRAP mM TE	mg/g GAE	mg/g RE
Tonda di Giffoni	13.2 ± 0.1 ^a^	23.3 ± 5.8 ^a^	155.3 ± 2.1 ^a^	27.2 ± 0.7 ^a^
Tonda Gentile Romana	13.1 ± 0.2 ^a^	23.1 ± 6.2 ^a^	153.3 ± 3.9 ^a^	27.8 ± 2.3 ^a^

Means ± SD (*n* = 9) followed by the same letter (^a^,^b^) within a column are not significantly different (*p* > 0.05).

**Table 2:** Proximate composition of hazelnut skins.

Varieties	Moisture	Ashes	Lipids	Proteins	Carbohydrates
	% (g/100 g)	g/100 g DW	g/100 g DW	g/100 g DW	g/100 g DW
Tonda di Giffoni	4.7 ± 0.2 ^a^	3.0 ± 0.02 ^a^	18.4 ± 0.1 ^b^	7.5 ± 0.2 ^a^	71.1 ± 0.3 ^a^
Tonda Gentile Romana	4.1 ± 0.1 ^a^	2.8 ± 0.02 ^a^	26.4 ± 0.1 ^a^	8.9 ± 0.3 ^a^	61.9 ± 0.2 ^b^

Means ± SD (*n* = 3) followed by the same letter (^a^,^b^) within a column are not significantly different (*p* > 0.05). DW (dry weight).

**Table 3:** Mass Spectral Characteristics of Phenolics Identified in Methanol Extract of Hazelnut Skins.

Compound	*m*/*z* (g/mol)	Tonda di Giffoni	DEV. SD	Tonda Gentile Romana	DEV. SD
Chlorflavonin	378.8	3.32 × 10^4^	2.29 × 10^4^	3.54 × 10^4^	7.78 × 10^3^
Flavonol 7-O-beta-D-glucoside	416.4l	1.03 × 10^6^	2.10 × 10^6^	9.68 × 10^4^	1.66 × 10^4^
Naringenin 7-O-beta-D-glucoside	434.4l	3.91 × 10^6^	3.92 × 10^6^	4.26 × 10^5^	7.01 × 10^4^
Leucocyanidin	306.27	3.45 × 10^6^	4.30 × 10^6^	1.89 × 10^6^	1.84 × 10^5^
Caffeic acid 3-glucoside	342.3	6.31 × 10^4^	3.19 × 10^4^	2.50 × 10^5^	3.23 × 10^4^
Epigallocatechin-(4beta->8)-epicatechin-3-O-gallate ester	746.6	6.55 × 10^5^	1.35 × 10^6^	1.02 × 10^5^	9.82 × 10^3^
Kaempferol 3-O-beta-D-glucosylgalactoside	610.5	8.40 × 10^5^	1.75 × 10^6^	5.26 × 10^4^	8.20 × 10^3^
Vitexin 2---O-beta-D-glucoside	593.5	6.18 × 10^5^	1.31 × 10^6^	8.82 × 10^4^	1.23 × 10^4^
Kaempferitrin	578.5	1.74 × 10^6^	2.80 × 10^6^	7.32 × 10^5^	2.05 × 10^6^
(-)-Catechin	290.27	1.93 × 10^7^	2.11 × 10^7^	6.31 × 10^7^	9.78 × 10^6^
Flavin	453.3	2.15 × 10^6^	2.40 × 10^6^	1.93 × 10^5^	1.56 × 10^4^
n-Propyl gallate	212.2	3.77 × 10^6^	3.75 × 10^6^	4.03 × 10^6^	7.47 × 10^5^
Methyl caffeate	194.18	1.01 × 10^6^	8.16 × 10^5^	1.44 × 10^6^	2.40 × 10^5^
Laricitrin	332.26	1.86 × 10^5^	4.56 × 10^4^	2.41 × 10^5^	5.02 × 10^4^
Syringetin	346.3	8.81 × 10^4^	1.32 × 10^4^	5.22 × 10^4^	6.87 × 10^3^
Apigenin	270.24	5.72 × 10^6^	1.31 × 10^6^	4.69 × 10^6^	7.24 × 10^5^
Kaempferol	286.24	1.06 × 10^6^	2.52 × 10^5^	2.13 × 10^6^	4.12 × 10^5^
Caffeate	180.16	3.67 × 10^5^	5.69 × 10^4^	6.77 × 10^5^	8.26 × 10^4^
3-Coumaric acid	164.16	2.46 × 10^6^	3.65 × 10^5^	2.56 × 10^6^	3.86 × 10^5^
Neochlorogenic acid	354.31	3.52 × 10^5^	6.34 × 10^4^	1.80 × 10^4^	2.90 × 10^3^
Salicylate	137.11	5.15 × 10^7^	9.23 × 10^6^	2.85 × 10^7^	5.83 × 10^6^
Vitexin	432.4	1.91 × 10^6^	3.80 × 10^5^	3.30 × 10^6^	6.04 × 10^5^
Astragalin	448.4	1.45 × 10^7^	1.48 × 10^6^	2.57 × 10^7^	2.67 × 10^6^
Quercetin 3-O-glucoside	464.4	1.23 × 10^6^	8.96 × 10^5^	3.20 × 10^6^	3.99 × 10^5^
Naringenin	272.25	4.83 × 10^6^	1.06 × 10^6^	5.77 × 10^6^	1.07 × 10^6^
Eriodictyol	288.25	3.73 × 10^6^	3.30 × 10^5^	7.03 × 10^6^	8.10 × 10^5^
Luteoforol	290.27	4.15 × 10^7^	6.45 × 10^6^	6.60 × 10^7^	1.01 × 10^7^
Ferulate	194.18	1.61 × 10^6^	3.04 × 10^5^	1.47 × 10^6^	2.32 × 10^5^
Sinapate	224.21	1.35 × 10^6^	7.66 × 10^5^	5.31 × 10^6^	7.88 × 10^5^
Sinapine	310.36	1.43 × 10^5^	3.07 × 10^4^	4.68 × 10^5^	8.39 × 10^4^
4-Hydroxystyrene	120.15	2.65 × 10^7^	3.53 × 10^6^	1.07 × 10^8^	1.34 × 10^7^
Salicylaldehyde	122.12	4.84 × 10^6^	8.03 × 10^5^	9.72 × 10^6^	1.19 × 10^6^
Isochorismate	224.17	3.77 × 10^5^	1.01 × 10^5^	1.48 × 10^5^	1.79 × 10^4^
Salicin	286.28	4.12 × 10^5^	4.67 × 10^5^	6.24 × 10^5^	9.80 × 10^4^
2-3-Dihydroxybenzoate	153.11	1.34 × 10^7^	1.53 × 10^6^	6.16 × 10^6^	8.50 × 10^5^
p-Coumaroyl quinic acid	338.31	2.58 × 10^6^	2.83 × 10^6^	1.93 × 10^5^	3.59 × 10^4^
Naringin chalcone	580.5	1.46 × 10^6^	1.12 × 10^6^	7.10 × 10^5^	1.14 × 10^5^
Kaempferide	300.26	8.11 × 10^4^	4.59 × 10^4^	3.24 × 10^5^	6.81 × 10^4^
Quercetin	302.2	8.58 × 10^6^	1.94 × 10^6^	5.10 × 10^6^	1.01 × 10^6^
Rutin	610.5	2.94 × 10^5^	1.08 × 10^5^	9.86 × 10^5^	2.05 × 10^5^
Myricetin	318.23	1.04 × 10^6^	1.92 × 10^5^	2.36 × 10^5^	3.51 × 10^4^

Means ± SD (*n* = 9). Data are represented as peak intensity.

**Table 4:** List of phenolic compounds quantified using LC–MS. Means ± SD (*n* = 9). Compounds followed by the same letter (^a^,^b^) within a row are not significantly different (*p* > 0.05).

Phenolic Compound	Tonda di Giffoni	Tonda Gentile Romana
	mg/100 g	mg/100 g
Quercetin	1.4 ± 0.07 ^a^	0.8 ± 0.01 ^b^
Rutin	0.4 ± 0.04 ^b^	0.8 ± 0.01 ^a^
Vitexin	2.5 ± 0.1 ^b^	3.9 ± 0.4 ^a^
Caffeic Acid	0.6 ± 0.05 ^a^	0.7 ± 0.05 ^a^
P-Coumeric Acid	0.7 ± 0.08 ^a^	0.7 ± 0.06 ^a^

## Data Availability

No new Data were created or analyzed in this study. Data sharing is not applicable to this article.

## Supplementary Materials

The following are available online at https://www.mdpi.com/article/10.3390/metabo11050296/s1, Figure S1: Q2 is an estimate of the predictive ability of the model and is calculated via cross-validation (CV). In each CV, the predicted data are compared with the original data, and the sum of squared errors is calculated. The prediction error is then summed over all samples (Predicted Residual Sum of squares or PRESS). For convenience, the PRESS is divided by the initial sum of squares and subtracted from 1 to resemble the scale of the R2. Good predictions will have a low PRESS or high Q2.

## Abbreviation

DPPH	2:2-diphenyl-1-picryl-hydrazyl-hydrate
DW	Dry Weight
ESI-MS	Electrospray Ionization- Mass Spectrometry
FDA	Food and Drug Administration
FDR	False Discovery Rate
FRAP	Ferric Reducing Antioxidant Power
GAE	Gallic Acid Equivalents
HAT	Hydrogen Atom Transfer
HILIC	Hydrophilic Interaction Liquid Chromatography
IT	Injection Time
LDL	Low-Density Lipoprotein
MetPA	Metabolomic Pathway Analysis
PCA	Principal Component Analysis
PCs	Principals Components
PDO	Protected Designation of Origin
PGI	Protected Geographical Indication
PLS-DA	Partial Least Squares Discriminant Analysis
RE	Rutin Equivalents
SET	Single Electron Transfer
TE	Trolox Equivalents
TEAC	Trolox Equivalent Antioxidant Capacity
TPTZ	Fe^3+^-2,4,6-tripyridyl-s-triazine
UHPLC	Ultra High-Performance Liquid Chromatography
