# Supplementary material for: Detection and Comparison of Bioactive Compounds in Different Extracts of Two Hazelnut Skin Varieties, Tonda Gentile Romana and Tonda Di Giffoni, Using a Metabolomics Approach
Source: Metabolites. 2021 May 5;11(5):296. doi: 10.3390/metabo11050296 (PMC8148165; doi:10.3390/metabo11050296)
Supplement: Supplementary file 1 [file metabolites-11-00296-s001.zip › metabolites-1195707-supplementary.pdf]

Supplementary file:

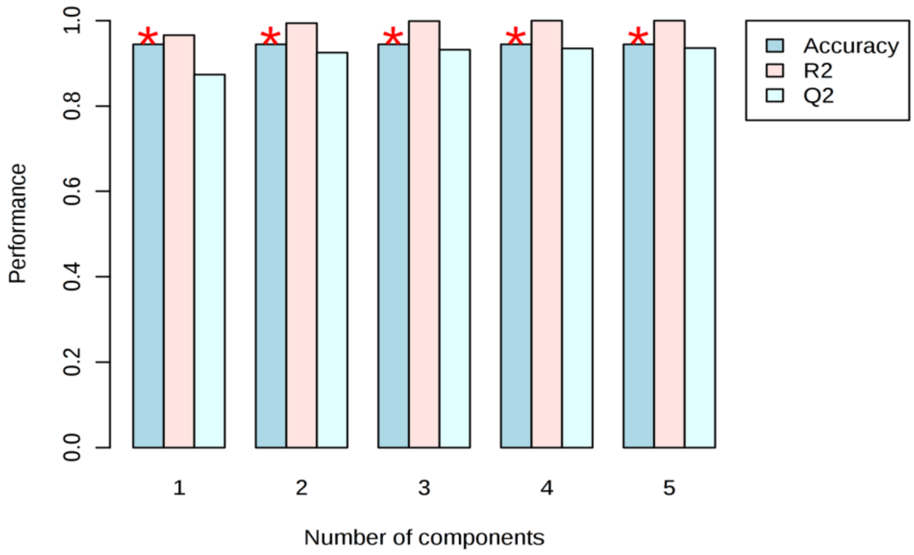

Measure	1 comps	2 comps	3 comps	4 comps	5 comps
Accuracy	0.94444	0.94444	0.94444	0.94444	0.94444
R2	0.96574	0.99439	0.99944	0.99969	0.99983
Q2	0.85139	0.92421	0.9325	0.93521	0.93582

Figure 1S: Q2 is an estimate of the predictive ability of the model, and is calculated via cross-validation (CV). In each CV, the predicted data are compared with the original data, and the sum of squared errors is calculated. The prediction error is then summed over all samples (Predicted Residual Sum of Squares or PRESS). For convenience, the PRESS is divided by the initial sum of squares and subtracted from 1 to resemble the scale of the R2. Good predictions will have low PRESS or high Q2.
